# Receptor Properties and Features of Cytokinin Signaling

**Published:** 2012

**Authors:** S.N. Lomin, D.M. Krivosheev, M.Yu. Steklov, D.I. Osolodkin, G.A. Romanov

**Affiliations:** Timiryazev Institute of Plant Physiology, Russian Academy of Sciences, Botanicheskaya Str., 35, Moscow, Russia,127276; Department of Chemistry, Lomonosov Moscow State University, Leninskie Gory, 1/3, Moscow, Russia, 119991; Belozersky Institute of Physicochemical Biology, Lomonosov Moscow State University, Leninskie Gory, 1/40, Moscow, Russia, 119992

**Keywords:** cytokinins, receptors, sensor histidine kinases, two-component systems, signal transduction

## Abstract

Cytokinins belong to one of the most important and well-known classes of plant hormones. Discovered over half a century ago, cytokinins have retained the attention of researchers due to the variety of the effects they have on the growth and development of vegetable organisms, their participation in a plant adaptation to external conditions, and the potential to be used in biotechnology, agriculture, medicine and even cosmetics. The molecular mechanism by which cytokinins function remained unknown for a long time. Things started to change only in the 21^st^century, after the discovery of the receptors for these phytohormones. It appeared that plants found ways to adapt a two-component signal transduction system borrowed from prokaryotic organisms for cytokinin signalling. This review covers the recent advances in research of the molecular basis for the perception and transduction of the cytokinin signal. Emphasis is placed on cytokinin receptors, their domain and three-dimensional structures, subcellular localization, signalling activity, effect of mutations, ligand-binding properties, and phylogeny.

## INTRODUCTION

**Fig. 1:**
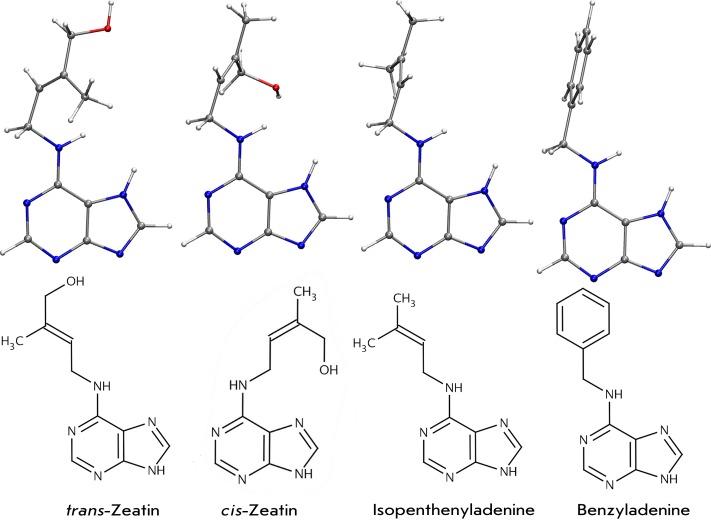
Structures of typical cytokinins. The most favoured conformations of cytokinins are shown in the upper line; their chemical structures are shown in the lower line.

Along with auxins, gibberellins, abscisic acid and ethylene, cytokinins belong to the group of classical plant hormones. Cytokinins were discovered by F. Skoog and co-workers in 1955 [1]. The hormone received its name because of the ability to activate *in vitro* division (cytokinesis) of plant cells. In terms of structure, natural cytokinins are adenine derivatives with a small substituent at the N ^6 ^ position ( *Fig. 1* ). Most cytokinins (e.g., zeatin, isopentenyladenine) have the isopentenyl group at this position; however, there can be an aromatic substituent (N ^6^ -benzyladenine, kinetin) as well. Certain synthetic derivatives of phenylurea (e.g., thidiazuron) also exhibit cytokinin activity. Cytokinins affect a number of physiological processes: they stimulate cell division and expansion, plastid differentiation, they retard the ageing process in leaves, activate metabolite inflow and shoot formation from calluses in culture [2–5]. Cytokinins are widely used in bioengineering and agricultural production to grow plant cell cultures in bioreactors, to carry out micropropagation (cloning) of cultivated plants, to obtain transgenic plants, to control plant sex, for cotton defoliation, etc. [4, 5]. Cytokinins participate in the inorganic nutrition of plants and in the formation of nitrogen-fixing root nodules, affect the cereal grain size (i.e., the crop capacity) and the plant resistance to adverse factors [6–8]. Cytokinins and related compounds have recently been finding increasing application in medicine and cosmetology; they are used as anti-tumor agents and inhibitors of neurodegenerative processes and as an active agent in liniments that prevent age-related changes in the skin [4, 9, 10].

During the past 15 years there has been substantial progress in elucidating the molecular mechanism of cytokinin action; sequencing of the genome of the model plant *Arabidopsis thaliana* played a significant role [11]. Discovery of receptors, the key components of hormone signal reception and transduction, was of particular significance. Four papers devoted to the identification and characterization of cytokinin receptors in *Arabidopsis thaliana * were published in 2001 [12–15]. A receptor named CRE1 (Cytokinin Response 1), or AHK4 (Arabidopsis Histidine Kinase 4), has been characterized. A mutation that manifested itself in a shortening of the Arabidopsis root in the absence of phloem ( *wooden* *leg* , or *wol* ) was identified before that. This mutation affects the same gene referred to as *WOL* [16]. In addition to the *CRE1/AHK4/WOL* gene, two of its paralogues which became known as *AHK2* and *AHK3* have also been identified in the Arabidopsis genome sequence [13, 14, 16, 17]. Thus, three cytokinin receptors have been identified in Arabidopsis; these receptors are transmembrane proteins with a similar structure and a molecular weight of over 100 kDa.

**Fig. 2:**
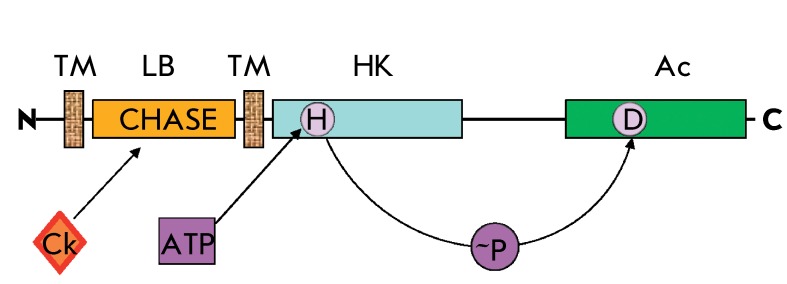
Domain structure of cytokinin receptor (exemplified by CRE1/AHK4 from Arabidopsis). Protein domains: TM – transmembrane; LB – ligand-binding (CHASE); HK – histidine kinase; Ac – acceptor; Ck – cytokinins; H – conserved histidine; D – conserved aspartate; N and C denote the N- and C-termini of the protein. The rightwards arrows indicate the sites for phosphorylation and the transfer of high-energy phosphates (~P).

This review is focused upon the major questions pertaining to cytokinin recognition and signalling, such as the domain structure of receptors, the biochemical basis of signal perception and transduction, subcellular localization, ligand-binding characteristics and the effect of mutations on receptor properties, the three-dimensional structure of receptors, and the emergence and evolution of receptors in plants.

## THE DOMAIN STRUCTIURE OF CYTOKININ RECEPTORS

Cytokinin receptors belong to the group of catalytic receptors. They have a complex multidomain structure ( *Fig. 2* ). The so-called CHASE domain (Cyclase/Histidine kinase Associated Sensory Extracellular) located at the N-terminus of a receptor molecule possesses hormone binding activity [18, 19]. There are two or more transmembrane domains at the two sides of this sensor domain. The last transmembrane domain is followed by a catalytic domain with histidine kinase activity. The core component of this region consists of a dimerization domain and the ATP/ADP binding phosphotransfer domain. The dimerization domain (A-domain) consists of two antiparallel helices that are adjacent to each other (two-stranded coiled-coils). The A-domains of two receptors can interact thus forming a four-helix bundle. According to current concepts, each histidine kinase subunit in the dimer is phosphorylated by the other one ( *in trans * reaction) [20]. The phosphotransfer domain contains a conserved site (H-box) of the general structure – **A** TV **SH** E **IR** TP – with the histidine residue being phosphorylated in its centre.

Four conserved motifs (N-, G1-, F-, and G2-boxes) participate in ATP binding. They probably participate in the catalysis and transfer of the phosphate moiety as well. The C-terminus of the receptor contains the receiver domain with the conserved acceptor aspartate residue in the sequence denoted as DD- **D-** K. Cytokinin receptors contain a pseudo-receiver domain which is structurally similar to the receiver domain but cannot receive a phosphate from the conserved histidine residue. The pseudo-receiver domain resides between the regions of histidine kinase and the receiver domains [21, 22]. The function of the pseudo-receiver domain has not been elucidated.

Thus, cytokinin receptors belong to the group of membrane sensor histidine kinases in terms of their general structure and are homologous to some other sensor proteins from plants (ethylene receptors and phytochromes) [22, 23].

## MOLECULAR BASIS OF CYTOKININ SIGNAL TRANSDUCTION

Cytokinin receptors are structural and functional relatives of sensor histidine kinases belonging to two-component signal transduction systems that are common among prokaryotes, and have also been found in a number of eukaryotes except for animals [20, 24]. A classical prokaryote two-component system consists of two proteins, namely, a sensor histidine kinase and a response regulator (usually a transcription factor). Under the influence of external factors the histidine kinase is activated and autophosphorylated. The high-energy phosphate then passes to the response regulator. In two-component systems, phosphate is transferred from the conserved histidine residue of one protein molecule (histidine kinase) to the conserved aspartate residue of another molecule (the receiver domain of the response regulator). This process is referred to as phosphorelay. The phosphorylation of the response regulator results in its activation, which in turn triggers transcription of a particular gene or gene set [25].

**Fig. 3:**
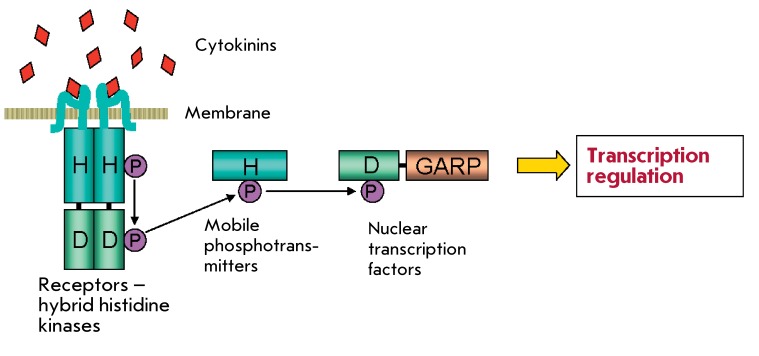
Scheme of cytokinin signal transduction based on the principle of multistep His-Asp-His-Asp phosphorelay. The phosphorylation of nuclear transcription factors (ARR-B-type response regulators) leads to their activation and subsequent alteration of the primary response gene transcription.

The scheme of signal transduction is more complex in the case of cytokinin perception, since the receiver domain is part of the sensor histidine kinase (a type of chimeric protein). The signal is transduced according to the principle of multistep (His-Asp-His-Asp) phosphorelay ( *Fig. 3* ). Hormone binding to the CHASE sensor domain yields phosphorylation of the conserved histidine residue in the resulting histidine kinase dimer. This phosphate moiety is subsequently intramolecularly transferred to the conserved aspartate residue of the receiver domain of histidine kinase and then transferred to the conserved histidine residue of the mobile low-molecular-weight protein-phosphotransmitter (HP), which permanently migrates between the cytoplasm and the cell nucleus [26]. When a phosphorylated phosphotransmitter enters the nucleus, it passes its high-energy phosphate to the conserved aspartate residue in the receiver domain of the response regulator. This protein (which is typically a transcription factor) is activated by phosphorylation and acquires the ability to regulate (typically, activate) the transcription of the primary response genes [27–29]. The cytokinin regulation of the biosynthesis of mRNA of the primary response genes is dependent only on a nontranscribed promoter; i.e., it occurs at the stage of transcription initiation [4].

## The features of cytokinin signaling in arabidopsis

The first cytokinin receptors were identified in Arabidopsis; their ability to recognize the hormonal signal has been confirmed in experiments with transformed bacteria and yeast. Expression of cytokinin receptors from plants in these unicellular organisms resulted in the emergence of a response to low (hormonal) cytokinin concentrations [12–14, 30, 31]. The *in vivo * role of these proteins as receptors has been verified by studying the insertional mutants of Arabidopsis. In general, the mutation in a single receptor does not result in any noticeable changes of plant phenotype. However, mutation in two and, in particular, all three receptors has serious effects. When all three receptors were inactivated, the triple mutant was insensitive to cytokinins and represented a sterile dwarf plant with reduced viability [32–34].

The receptors functionally complement each other, although they are not redundant in a number of processes. The CRE1/АНК4 receptor is mainly expressed in roots, whereas АНК3 prevails in leaves. In accordance, the effects of cytokinin on the aerial and underground parts of a plant depend to a larger extent on the AHK3 and CRE1/АНК4 receptors, respectively [4]. Five typical phosphotransmitters (AHP) and 22 response regulators have been identified among the elements of the two-component system in Arabidopsis. Phosphotransmitters are small proteins up to 17 kDa [35]. Similarly to receptors, AHP proteins are redundant and participate in the transduction of the cytokinin signal additively; the mutant with respect to all five genes exhibited abruptly reduced sensitivity to cytokinins and phenotypically resembled the receptor triple mutant [36–39]. AHP 1, 2, 3 and 5 play the key role in cytokinin signal transduction. According to current concepts, AHP proteins permanently migrate between the nucleus and the cytoplasm; the pattern of their localization is independent of phosphorylation [26, 35, 40].

AHP6 is another Arabidopsis protein that structurally resembles the phosphotransmitter. However, this protein belongs to pseudo-AHPs since it does not contain the conserved histidine residue required for the phosphorelay. The AHP6 binds to both receptors or response regulators inhibiting their interaction with typical phosphotransmitters and thus acting as a negative regulator of cytokinin signal transduction [41].

Arabidopsis response regulators can be classified into three groups (A, B, and C); there is also a group of pseudo-regulator proteins [42]. The B-type response regulators which contain both the phosphorylated N-terminal receiver domain and a special B-motif including the DNA-binding GARP-domain and the glutamine-rich domain are the real transcription factors [43–46]. Due to the nuclear location signals (NLS) B-type response regulators are localized in the nucleus. The total number of *ARR-B* genes is 11; however, B-type response regulators are not identical in terms of cytokinin signal transduction. The *ARR1, 10* and *12 * genes play the key role: the triple mutant with knocked out genes is phenotypically similar to the cytokinin receptor triple mutant [47–49]. The expression of the genes of B-type response regulators is not regulated by cytokinins [28, 29, 50, 51]. It should be mentioned that direct evidence of the interaction between the proteins that are components of the signal transduction circuit and their ability to donate and accept phosphate according to the scheme shown in *Fig. 3* have been obtained [13, 36, 38].

As opposed to ARR-B, the genes of A-type response regulators (ARR-A) can be promptly activated by cytokinins and belong to the primary response genes for these hormones [27–29, 52]. ARR-A consist of the typical receiver domain and a small C-terminal fragment. The A-type response regulators may accept phosphate from phosphotransmitters similar to the B-type regulators; however, they cannot induce the typical transcription response.

A body of observations allows to conclude that ARR-A act as negative regulators of signal transduction, the conserved aspartate residue being required to implement their inhibitory effect [53–55]. The multiple mutant with respect to the genes of A-type response regulators is characterized by increased sensitivity to cytokinin. It is assumed that the A-type response regulators are capable of suppressing cytokinin signal transduction from the AHP proteins by competing with the B-type regulators for the high-energy phosphate. Thus, the participation of ARR-A in the system of cytokinin signal transduction provides negative feedback. Although the structure of C-type response regulators is similar to that of ARR-A, they are not induced by cytokinins and seem not to play a significant role in cytokinin signal transduction [42, 56]. In the absence of cytokinin the CRE1/AHK4 receptor acts as phosphatase and removes a phosphate group from the AHP proteins, thus deactivating signal transduction from the other cytokinin, receptors too [57]. In general, a large number of studies carried out using Arabidopsis plants have persuasively demonstrated that the cytokinin signal is transduced via the two-component pathway, with hybrid histidine kinases acting as receptors.

## SUBCELLULAR LOCALIZATION OF RECEPTORS

The cytokinin receptor is an integral transmembrane protein with the CHASE domain located to one side of the membrane and the histidine kinase and receiver domains located to the other side. Cytokinin receptors were believed to be localized on the plasma membrane; it was assumed evident that the CHASE domain has to be localized extracellularly, whereas the remaining part of the protein has to be intracellular. This assumption was partly based on the computational prediction of subcellular localization [12, 14, 16] and on the analogy with a bacterial cell, where the CHASE domain of sensor proteins is extracellular (this fact is evident from the domain name). This belief was further bolstered when the localization of the cytokinin receptor in the plasmalemma revealed by expression of the * АНК3-GFP* construct in Arabidopsis protoplasts was reported [58]. The localization of cytokinin receptors on the plasma membrane assumes that the cytokinin signal enters the cell from the environment due to extracellular cytokinins. On the other hand, it was demonstrated by determining the pH-dependence of cytokinin binding to receptors that the binding is optimal in neutral and alkalescent media, which are typical of the cytoplasm, and that it decreases abruptly under acidified conditions, which are typical of the extracellular space (the apoplast) [59]. This fact attests on the contrary to the intracellular localization of the receptor. Therefore, studies of the subcellular localization of cytokinin receptors were continued.

Three articles claiming that the receptors (or at least their majority) are localized inside the cell on the membranes of the endoplasmic reticulum (ER) have recently been published [60–62]. Sites of ^3^ Н- *trans* -zeatin high-affinity binding in the fraction containing membranes (microsomes) but not in fractions containing mitochondria or chloroplasts have been revealed in experiments with subcellular organelles [60]. After the microsomes had been separated in an aqueous two-phase polymer system into the plasmalemma and endomembranes, it turned out that the high-affinity sites were mostly confined to the endomembrane fraction both in Arabidopsis [61] and corn [60]. Taking into account the predominance of endomembranes in the cell, it was assessed that over 90% of the hormone binding sites are localized intracellularly.

By studying the localization of the Arabidopsis receptor-fluorescent protein fusions expressed in tobacco leaves [61, 62] and the corn receptor ZmHK1 in protoplasts from corn leaves [60] it has been demonstrated that fluorescence distribution corresponds to the endoplasmic reticulum network. For the AHK3 receptor, the fluorescence pattern coincided with the pattern for the ER marker but not the plasmalemma marker [61, 62]. In addition, the AHK3 protein was *in vivo * glycosylated at the sites that are sensitive to glycosidase endoH, which attests to localization in ER [62]. The same glycosylation was recorded in control experiments for the ethylene receptor ERS1 integrated into ER [63, 64], whereas the potential endoH-sites in histidine kinase AHK1 localized in the plasmalemma were not glycosylated [62].

It should be mentioned that the intracellular localization of cytokinin receptors, which was revealed via fluorescence, was observed under various conditions of expression of the inserted genes using promoters of different strengths. However, the most convincing result was obtained by analysis of the localization of the receptors expressed under natural conditions. This approach was implemented via immunoblotting with antibodies against the corn receptor ZmHK1. The membrane fractions obtained upon separation in a sucrose gradient in the absence or presence of magnesium cations were analyzed [60]. In the absence of magnesium, ribosomes dissociate from the ER, resulting in the shift of the ER towards the top of the gradient. This shift is not observed if magnesium is present in the medium. This effect referred to as the Mg-shift is typical of ER but not the other membranes unbound to the ribosomes. It was demonstrated by the analysis of fractions from corn cells that the ZmHK1 protein undergoes a Mg-shift and is co-localized with the ER marker protein (BiP) [60].

The stable Arabidopsis transformants expressing AHK2 or AHK3 receptor genes under their own promoters and with the Myc peptide at the C-terminus of the protein were obtained . The expression of these constructs compensated for the phenotype of the *ahk2 ahk3* double mutant of Arabidopsis, attesting to the functionality of these modified receptors. The typical Mg-shift and correlation with the ER marker were also revealed when analyzing the membrane fractions via immunoblotting with anti-Myc antibodies [61].

All these data allow one to conclude that cytokinin receptors are mostly localized in the endoplasmic reticulum. Along with the data pertaining to the ability of ER-localized receptors to bind cytokinins and the pH-dependence of this binding typical of cytoplasmic proteins, this result may attest to the fact that cytokinin signal perception occurs mainly inside the cell and that intracellular cytokinins play the key role in this process. However, the presence of a small number of receptors on the plasma membrane should not be left without consideration. These receptors can be responsible for the perception of the signal from extracellular cytokinins. Further research is needed to assess the functional properties of each pool of cytokinin receptors.

## ligand-binding properties of receptors

Along with gibberellines, cytokinins are represented by a variety of isoforms in plants ( *Fig. 1* ); among those *trans* - and *cis* -zeatins, isopentenyladenine, dihydrozeatin (bases), their N ^9^ -ribosylated derivatives (ribosides) and N ^9^ -riboside phosphate derivatives (nucleotides) are prevalent. Aromatic cytokinins such as N ^6^ -benzyladenine and its derivatives, topolin, etc. occur as well [4, 5, 65]. Cytokinins migrate within a plant along transport channels: in the upward direction from the root into the shoot via xylem and in the downward and other directions via the phloem. Cytokinin compositions in the xylem differ from those in the phloem: *trans* -zeatin-type cytokinins (mostly, *trans* -zeatin-riboside) are the prevailing isoforms in the xylem, whereas isopentenyl-type cytokinins are prevalent in the phloem [66–68].

The physiological role of each cytokinin isoform is determined by its affinity to the receptor; therefore, the investigation of cytokinin–receptor interaction and ligand specificity of the receptors is of high importance. The ligand-binding properties of cytokinin receptors have been studied mostly using heterologous model systems upon expression of the receptor genes in transformed bacterial ( *Escherichia coli* ) or yeast cells. Plant receptors turned out to be capable of functional replacement of mutant sensor histidine kinases with a similar (hybrid) structure in these unicellular organisms [12, 13, 30].

Both functional tests [13, 15, 30, 69, 70] and hormone-receptor binding assays [59, 60, 71, 72] have been carried out based on the aforementioned model systems. In general, as was expected, the affinity of the hormone to the receptor positively correlated with the hormone’s ability to induce a biological response [59, 71]. *trans* -Zeatin is one of the most active ligands for most of the receptors studied; *К*_d _ of the hormone-receptor complex varies within a range of 1–10 nM. Such values of the constants are typical of high-affinity hormone-receptor interactions. Let us note that these values of the constants are close to the measured concentrations of *trans* -zeatin in living plants [34, 66, 68, 73]. A Scatchard analysis has revealed the single receptor-ligand binding site without any signs of cooperative interaction [59, 74]. Meanwhile, natural (N ^6^ -adenine derivatives) and synthetic (thidiazuron, a phenylurea derivative) cytokinins bound to the same receptor site [59].

**Table 1:** Rows of cytokinin affinity for the receptors from Arabidopsis and corn

Species	Receptor**	Cytokinin affinity rows**
*Zea mays *	ZmHK1	iP ≥ BA >> tZ ≥ cZ >> DZ >> Ade
*Arabidopsis thaliana *	CRE1/AHK4	iP ≥ tZ > BA > DZ > cZ >> Ade
*Zea mays *	ZmHK2	tZ ≥ iP > DZ > BA > cZ >> Ade
*Arabidopsis thaliana *	AHK3	tZ > DZ > iP > cZ > BA >> Ade
*Zea mays *	ZmHK3a	iP > tZ > BA > cZ >> DZ >> Ade
*Arabidopsis thaliana *	AHK2	iP > tZ > BA > cZ > DZ >> Ade

* Orthologous receptors are grouped pairwise.

** Cytokinins: iP – isopentenyladenine; BA – N ^6^ -benzyladenine; tZ – *trans* -zeatin; cZ – *cis* -zeatin; DZ – dihydrozeatin. Ade – adenine.

Yet, the receptors differ in their preference of cytokinin isoforms [59, 60, 75]. The Arabidopsis receptors CRE1/AHK4 and АНК2 have the same high affinity to *trans* -zeatin and isopentenyladenine and a considerably lower affinity to dihydrozeatin. On the contrary, the AHK3 receptor is characterized by a relatively high affinity to dihydrozeatin and a lower affinity to isopentenyladenine. All three Arabidopsis receptors are capable of binding, though with low affinity, *cis* -zeatin, too. Cytokinin glucosylation at the N3 or N7 nitrogen atoms and at the oxygen atom of the side chain blocks the hormone-receptor binding [30, 59].

The ligand specificity of cytokinin receptors has also been studied for corn, a monocotyledonous plant, three receptors of which are orthologous to those of the dicotyledonous plant Arabidopsis: ZmHK1 orthologous to CRE1/AHK4; ZmHK2 orthologous to АНК3; and ZmHK3 orthologous to АНК2 [76]. The corn receptors were partly similar to, partly different from, their Arabidopsis counterparts [60, 76]. In general, the order of relative ligand activity turned out to be rather similar for corn and Arabidopsis orthologues ( *Table 1* ). Whereas isopentenyladenine exhibited higher activity than *trans* -zeatin with respect to ZmHK1 and ZmHK3, the opposite was observed for ZmHK2. A stronger difference between corn receptors was observed upon their interaction with dihydrozeatin: the affinity of ZmHK2 to this cytokinin is more than two orders of magnitude higher compared to its affinity to ZmHK1 and ZmHK3. A relatively high affinity to *cis* -zeatin is a characteristic feature of corn receptors, ZmHK1 demonstrating almost identical affinities to *trans* - and *cis* -zeatins. This feature of corn receptors is in accordance with an increased concentration of *cis* -zeatin in this plant species [77, 78].

**Fig. 4:**
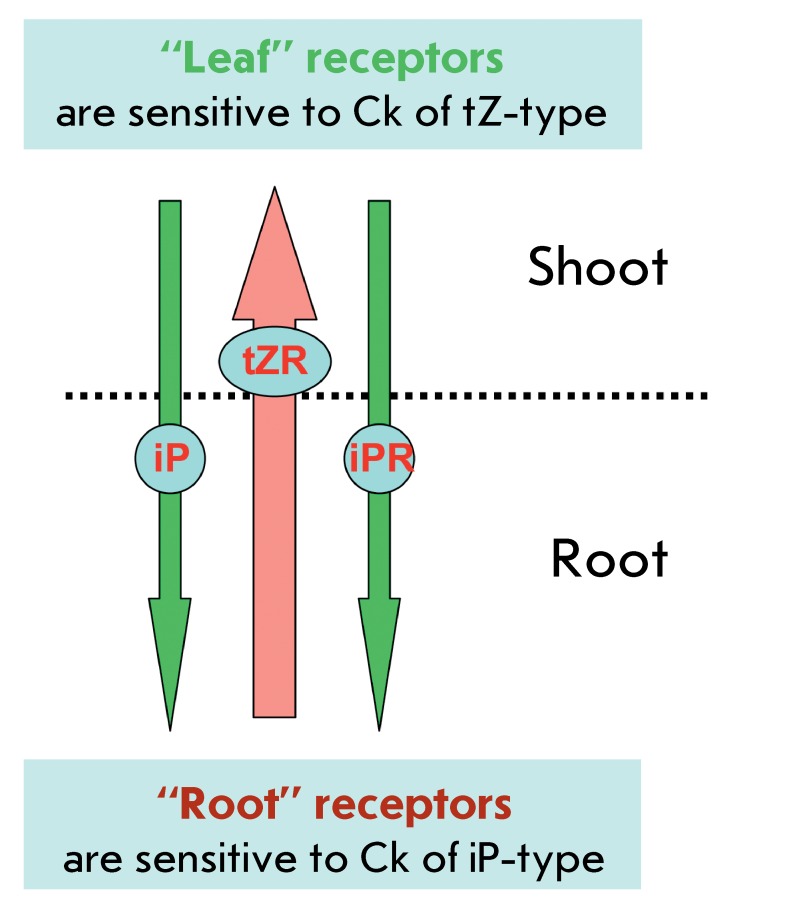
A model for long-distance cytokinin action. The arrow in the middle denotes the translocation of cytokinins (Ck) of *trans* -zeatin type from the root to the shoot via xylem. The lateral arrows denote the translocation of cytokinins of the isopentenyladenine type from the shoot to the root via phloem.

The regularities of the receptor preferences to certain ligands can be interpreted with allowance for their possible role in long-range signalling in plants. The Arabidopsis receptors AHK3 and their orthologues ZmHK2 in corn are mainly expressed in shoots and control the metabolic processes occurring in leaves. These receptors are “tuned” primarily to *trans* -zeatin-type cytokinins; i.e., to the cytokinins transported to the shoot from the roots. In turn, the CRE1/AHK4 and ZmHK1 receptors that are prevalent in roots actively respond to isopentenyladenine, the major cytokinin in phloem, which is translocated from the shoot to the roots with the phloem sap ( *Fig. 4* ). Thus, signal exchange can occur between different parts and organs of a plant organism, when the cytokinin signals of a remote organ turn out to be more significant for the cell compared to the signals from the closer located tissues [4, 59, 79].

## effect of mutations on receptor activity

The identification of the Arabidopsis mutation named *wooden leg * ( *wol* ) resulted in the discovery of cytokinin receptors. The mutant plants were different from the wild-type plants by a shorter length and a disturbed development of the vascular system of the main root. The latter consisted of protoxylem only (metaxylem and phloem have not been developed); the total number of cells was significantly lower. Moreover, the plants had no lateral roots and exhibited enhanced formation of adventitious roots. The phenotypic manifestation of this mutation was first described in 1995 [80].

The *wol* mutation was subsequently found to be localized in the gene of sensor histidine kinase CRE1/AHK4 and to consist in the substitution of threonine 278 (threonine 301 according to the modern numeration) with isoleucine in the hormone-binding CHASE domain [12, 15, 16]. The *wol* -2 and *cre1-1* mutations were later obtained via chemical mutagenesis. These mutations consisted in replacing leucine 529 with isoleucine [82] and glycine 490 with aspartate [12], respectively. All these mutations also resulted in the typical *wol* phenotype caused by underdevelopment of the vascular system due to a reduced number of meristem initial cells because of blockage of cell division [16]. The defects in the vascular system impeded auxin transport to the pericycle; as a result, lateral roots were not formed. Meanwhile, the disturbance in the vascular system of the main root resulted in auxin accumulation in the hypocotyl bottom region, which in turn stimulated the formation of adventitious roots. It is of interest to note that normal development of the vascular system in adventitious roots, as opposed to the main root, was observed in the *wol-3* mutants [82].

The radioligand-binding technique demonstrated that the receptor loses its cytokinin-binding capacity upon *wol * mutation [15]. However a stop codon introduced to the mutant *CRE1/AHK4 * gene recovered the wild-type phenotype in *wol * plants [57]. Therefore, it was reasonable to assume that the mutant receptor CRE1/AHK4 not only stops participating in cytokinin signal transduction but also suppresses the transduction of this signal from the other receptors, AHK2 and AHK3. Some bacterial histidine kinases have been ascertained to possess phosphatase (in addition to kinase) activity, resulting in dephosphorylation of phosphoproteins [83]. It has been demonstrated in *in vitro * experiments and in experiments on transgenic yeasts that CRE1/AHK4 also has a constitutive phosphatase activity, whereas its histidine kinase activity is manifested only in the presence of cytokinins [57]. Thus, the *wol * mutation which makes CRE1/AHK4 incapable of cytokinin binding blocks its histidine kinase activity, whereas phosphatase activity is retained. Consequently CRE1/AHK4 harboring the *wol * mutation dephosphorylates the phosphotransmitter proteins phosphorylated with the AHK3 and AHK2 receptors, thus blocking the cytokinin signal transduction. CRE1/AHK4 predominates in root cells [17, 32]; therefore, the *wol* mutant phenotype is mainly manifested in roots.

The same mutations in the CHASE domain of AHK3 and AHK2 receptors have not resulted in the emergence of the *wol* -like phenotype [31]. This attests to the fact that the AHK2 and AHK3 receptors do not have phosphatase activity.

Thus, a number of mutations in the CRE1/AHK4 receptors resulting in the emergence of the typical *wol* -phenotype have been revealed. It has been demonstrated that the receptor, upon all these mutations, no longer transduces cytokinin signals despite the fact that only the *wol* ( *wol-1* ) mutation is localized in the CHASE domain. The *wol-3* mutation is localized in the region between the second transmembrane and histidine kinase domains; the *wol-2* and *cre1-1* mutations are localized in the histidine kinase domain.

In general, the analysis of mutations in cytokinin receptors has enabled to confirm and refine our understanding of the roles of the defined parts of receptors. The isolated CHASE domain with the adjacent transmembrane domains retains the ability to high-affinity cytokinin binding, whereas the receptor without the CHASE domain lacks such ability [84]. Hormone-receptor binding was also suppressed by the other mutations in this domain [84]. Thus, the role of the CHASE domain as a hormone-binding one is beyond question.

Although the mutations in the CHASE domain disturbed receptor functioning in the absolute majority of cases, a mutation (called *ore12-1* ) in this domain resulting in constitutive activity of histidine kinase was found in the AHK3 receptor [58]. Upon this mutation proline 243 located in the middle of the CHASE domain was replaced by serine. It was assumed that this substitution of amino acids could result in an alteration of the CHASE domain structure similar to that caused by cytokinin binding [58].

Conserved histidine and aspartate residues which undergo phosphorylation during the signal transduction are known to play a special role in the molecules of sensor hybrid histidine kinases. Substitution of these residues (His482Gln and Asp996Asn) resulted in a loss of both histidine kinase activity and the ability of CRE1/AHK4 to respond to cytokinins [12]. The substitution of Asp996Asn also resulted in a total loss of phosphatase activity, whereas substitution of histidine caused only a slight reduction in the activity [57]. Note that the His482Gln replacement did not alter the cytokinin-binding capacity of the receptor [84].

A number of mutations in CRE1/AHK4 have been obtained using PCR: Gly435Cys, Phe436Ser, Met447Thr in the second transmembrane domain; Val471Ala in the region between the second transmembrane and histidine kinase domains; and Met494Leu in the histidine kinase domain. All these mutations were localized in a short region of approximately 60 amino acid residues between the ligand-binding domain and the conserved histidine residue which plays a significant role in protein autophosphorylation [31]. These mutations led to the emergence of constitutive histidine kinase activity in CRE1/AHK4; i.e., this receptor acquired the ability to send a signal whether cytokinins were present in the media or not. Meanwhile, the mutant receptors retained their cytokinin-binding capacity, which has been confirmed in experiments of tritium-labelled isopentenyladenine binding by these receptors within the membranes of *Sсhizosaccharomyces pombe* . It is interesting to note that the CRE1/AHK4 receptor with the Phe436 mutations retained its constitutive histidine kinase activity even after the *wol* mutation was additionally introduced, despite losing its cytokinin-binding capacity. Thus, in the presence of these constitutive mutations the cytokinin-binding capacity of the receptor plays no role in signal transduction [31].

The introduction of mutations into the same regions of the other cytokinin receptors may also yield the same results. For instance, substitutions of conserved hydrophobic amino acids in the AHK2 (Ile586Ala) and AHK3 (Val449Ala) receptors, similar to the Val471Ala substitution in the CRE1/AHK4 receptor, resulted in the emergence of constitutive histidine kinase activity in the receptors [31]. The replacements of amino acids in the second transmembrane domain and in the downstream region could result in conformation changes in the protein molecule similar to those emerging upon cytokinin-receptor binding, thus stimulating histidine kinase activity in the absence of hormone.

Based on the structure of the cytokinin receptor, it is reasonable to expect that mutations removing the receiver domain or disturbing its structure will result in receptor inactivation. Indeed, plants *A. thaliana* carryingmutations in the CRE1/AHK4 receptor gene (called *cre1-3* and *cre1-7* ) where the triplets encoding Trp1026 and Gln475, respectively, were replaced with stop codons have been obtained [85]. It is evident that these mutations result in the synthesis of a truncated receptor lacking the entire or part of the receiver domain. In the *cre1-* 6 mutant, the replacement of nucleotides resulting in Gly493Ala substitution apparently leads to splicing disturbances and to the formation of the truncated receptor. Thr1008Ile and Ala1032Thr substitutions occurred in the mutants *cre1-4 * and *cre1-9* , respectively. They resulted in the formation of full-size proteins carrying mutations in the receiver domain [85]. The response to phosphate starvation, which is suppressed by cytokinins under normal conditions, was examined in the resulting mutant plants. As opposed to the controls, the mutant plants almost did not respond to cytokinin in this biotest. Thus, mutations leading to the formation of truncated CRE1/AHK4 receptors and mutations in the receiver domain resulted in the suppression of cytokinin sensitivity of plants in the phosphate starvation biotest [85].

Similar mutations in the MtCRE1 cytokinin receptor have been obtained and studied in lucerne * Medicago truncatula* [86]. These mutations affect the histidine kinase domain of the receptor. In the case of the *mtcre1-1 * mutation, the Trp573-encoding triplet localized in the middle of the domain was substituted with a stop codon, resulting in the formation of a truncated protein. The mutation in *mtcre1-2* consisted in the replacement of Thr642Ile in the conserved G2 motif of the domain. Upon mutation in *mtcre1-3* , the substitution Gly545Glu was localized in the variable region of the domain. It has been demonstrated in the biotest for root growth suppression that the *mtcre1-1 * and *mtcre1-2* mutants, as opposed to the *mtcre1-3* mutant, lose their sensitivity to cytokinin. Nodule formation upon exposure to symbiotic bacteria was disturbed in the *mtcre1-1 * and *mtcre1-2 * mutants [86]. All these facts underscore the significant role of each conserved domain in the normal functioning of the receptor.

## Three-dimensional structure of the receptor

To understand the structural and functional features of the receptor, it is important to know the three-dimensional structure of the protein under study. X-ray crystallography is the most common technique for studying the three-dimensional structure; a protein monocrystal is required to carry out this type of analysis. However, crystallization of cytokinin receptors is complicated since they are high-molecular-weight transmembrane proteins. Therefore, thus far structural studies have not been completed for any of these receptors.

It is more realistic to shed light on the structure of a domain of the receptor. Research in this area has been done for the ligand-binding [79, 87–89] and receiver [90, 91] domains. An attempt to predict the tertiary structure of the CHASE domain of the CRE1/AHK4 receptor was made back in 2004 [87]. Homology modeling of the CHASE domain based on the X-ray structures of the ligand-binding regions of the sensor histidine kinases from bacteria * E. coli* (PDB ID: 1OJG) and *Klebsiella pneumoniae* (PDB ID: 1P0Z) was used in this study. Molecular docking studies of the cytokinins *trans* -zeatin and kinetin into the putative binding site of this model were subsequently carried out. The results showed that the CHASE domain corresponds to the so-called PAS-type domain; the binding site covered the entire cytokinin molecule. A number of amino acid residues responsible for protein-ligand binding have been identified [87] (including Thr278, whose substitution with Ile - the *wol* mutation - resulted in receptor inactivation). However, the proposed model turned out to be generally incorrect presumably due to the too-distant relationship between the template proteins and the CHASE domain of CRE1/AHK4.

The investigation of the tentative structure of the hormone binding site in the CHASE domain was continued using the evolutionary proteomics approach; i.e., the search for the conserved amino acids of the CHASE domain required for ligand recognition and binding [84]. Several amino acid residues that may participate in the interaction with hormone have been found; five of them were substituted with alanine in the CRE1/AHK4 receptor. Upon expression of these mutant receptors in *E. coli* , two out of five substitutions (Phe281Ala and Thr294Ala) led to complete elimination of the hormone-binding capacity of the receptor. In two cases (Trp221Ala and Arg282Ala) binding decreased considerably as compared to the intact CRE1/AHK4 receptor. The Lys274Ala mutation had no effect. It has been noted that most efficient mutation sites are localized near the predicted central β-sheet structures of the domain, which assumes that these β-strands play a significant role in hormone binding. These results were essentially confirmed by subsequent identification of the X-ray structure of the CHASE domain in complex with the hormone [89]: the amino acid residues Thr294, Phe281, and Arg282, indeed, were in contact with cytokinin, whereas Lys274 did not form direct contacts with the hormone.

**Fig. 5:**
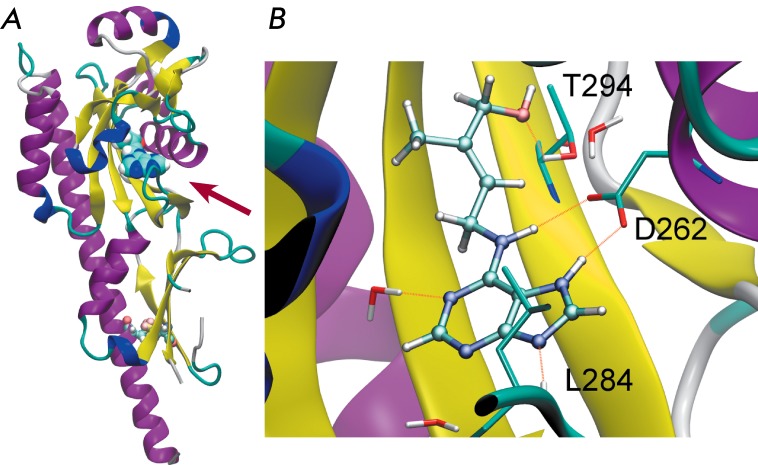
Three dimensional structure of the CHASE domain of the Arabidopsis cytokinin receptor CRE1/AHK4. The general view (A) and structure of the binding site with a *trans* -zeatin molecule (B). Cytokinin molecule is shown in spacefill representation, cystine bridge is shown as the ball-and-stick model. The arrow indicates the location of the bound cytokinin.

Decisive success in determining the three-dimensional structure of the CHASE domain was achieved in 2011, when a research team from the Salk Institute (USA) obtained a crystal of the CHASE domain of the CRE1/AHK4 receptor suitable for X-ray crystallographic study [89]. This allowed to determine the structure of the ligand-binding CHASE domain of the CRE1/AHK4 receptor in complex with various cytokinins (PDB ID: 3T4J, 3T4K, 3T4L, 3T4O, 3T4Q, 3T4S, 3T4T; resolution 1.53–2.30 Å). According to the data obtained ( *Fig. 5* ), the N-terminus of the CHASE domain forms a long α-helix neighboring two PAS domains connected by helical linkers. The β-strand closer to the C-terminus of the PAS domain is covalently linked to the N-terminal α-helix via a disulfide bridge, which makes the domain structure more rigid and compact. It is interesting to note that similar tertiary structures of the sensor domains were previously identified in the histidine kinases of certain bacteria ( *Bacillus subtilis* , PDB ID: 2FOS, 4DBJ; *Sinorhizobium meliloti* , PDB ID: 3E4P; *Shewanella oneidensis* , PDB ID: 3LIC) despite the low similarity between the sequences of the bacterial receptors and CRE1/AHK4 [92]. The sensor domains of both CRE1/AHK4 and their bacterial homologues crystallized in the form of homodimers. It has been ascertained that for cytokinin recognition CRE1/AHK4 uses the PAS domain located at a significant distance from the membrane. The ligand-binding cavity of the receptor completely embraces the ligand, as shown for a number of the best-known cytokinins: isopentenyladenine (3T4J), N ^6^ -benzyladenine (3T4K), *trans* -zeatin (3T4L), and kinetin (3T4S); differences between the structures of the receptor CHASE domain in complex with various hormones were negligible. The “floor” component of the cytokinin-binding site is formed by the central β-sheet of the PAS domain and is lined by small hydrophobic amino acid residues. Substitutions of these residues with bulkier amino acids block the cavity for cytokinin binding, thus inactivating the receptor. That just occurs upon the most common mutation *wol,* where the small Thr278 residue is substituted with Ile having a bulkier side chain. Two short β-strands form the hydrophobic “ceiling” of the active site. The hydrogen bonds are formed between the adenine component of cytokinin and the Asp262 residue (these bonds play the crucial role in binding), Leu284, Tyr250, and Thr286. The two latter hydrogen bonds are mediated by water molecules, which in turn interact with cytokinin atoms. The remaining residues participate in hydrophobic interactions with both the adenine- and, in particular, the tail component of cytokinin ( *Table 2). * The total number of amino acids forming the ligand binding pocket is approximately 20 ([89] and Hothorn M., personal communication).

**Table 2:** Amino acid residues forming the cytokinin binding site of the CRE1/AHK4 receptor.

Region in contact with hormone	Amino acid residues in the CHASE domain cavity surrounding the bound N^6^-isopentenyladenine*												
Adenine component	G200	M226	V248	Y250	L251	D262	F281	R282	L283	L284	T286	V292	A322
	1	1	2	H*H*	3	HH	1	1	3	H; 2	H*	3	3
Tail component	A202	A204	V241	M256	I266	T294	Y318	G320	G321				
	3	3	3	3	3	3	3	3	3				

* 1, 2, and 3: relative strength of hydrophobic interactions between iP and AHK4/CRE1; H, and HH: 1–2 hydrogen bonds, H*: hydrogen bond via water molecule

The amino acids that play a significant role in binding appeared to be appreciably conserved in different cytokinin receptors; substitution of these conserved residues in the CHASE domain of CRE1/AHK4 typically resulted in receptor inactivation [89].

In plants, cytokinins can be glycosylated at nitrogen atoms of the adenine moiety, whereas the OH group of the isoprenoid component of the ligand can be acylated or glycosylated. As previously mentioned, all these modifications render cytokinins inactive [30, 59]. The X-ray structure of the receptor supports these results, since the limited volume of the ligand-binding cavity is not sufficient to enclose cytokinins carrying additional glycosyl or other groups.

As opposed to *cis* -zeatin, *trans* -zeatin forms an additional hydrogen bond with Thr294 via the OH group of the side chain. This fact makes it clear why CRE1/AHK4 binds *trans* -zeatin with higher affinity than *cis* -zeatin. The binding mode for cytokinins carrying more bulky aromatic tail components was demonstrated by the example of kinetin and benzyladenine. The furfuryl moiety of kinetin, similar to the isoprenyl group of *trans* -zeatin, forms a hydrogen bond with Thr294 in the case of kinetin via water molecule. Using thidiazuron (3T4T structure) as an example it has been confirmed that the CRE1/AHK4 receptor uses the same site for binding synthetic and natural cytokinins, synthetic cytokinins forming hydrogen bonds with the same amino acids as cytokinins - N ^6^ -adenine derivatives.

The general principles used to design compounds with cytokinin activity are as follows: such compounds need to have a planar ring structure occupying the “adenine” part of the ligand binding cavity, with a linker that is capable of forming hydrogen bonds with Asn262 and attaching a small planar aliphatic or aromatic tail group [89].

## phylogenetic analysis of cytokinin receptors

Until recently, cytokinin receptors had been studied in detail only in two plant species (although phylogenetically rather distant): Arabidopsis and corn. Therefore, it became of interest to elucidate the features of the cytokinin perception apparatus in other plants species and trace back the formation of the cytokinin signalling system during plant evolution. Such research became possible thanks to the complete genome sequencing of a number of plant species.

The results of the phylogenetic analysis of a number of genomes has enabled researchers to conclude that the pathway of perception and transduction of the cytokinin signal based on two-component system emerged in metaphytes after transition to terrestrial life as one of the aspects of their biochemical adaptation to new living conditions [93]. The genes encoding sensor histidine kinases with the CHASE domain and A-type response regulators in the genomes of studied species of lower and higher plants have been found starting from mosses and spikemosses. In higher organized plants the number of components of the cytokinin signalling system is usually higher compared to that in more primitive plants. In particular, this applies to phosphotransmitters and response regulators. It has been noted that the cytokinin receptors of all flowering plants analyzed fall into three individual branches of the phylogenetic tree, corresponding to the Arabidopsis receptors CRE1/AHK4, AHK3, and AHK2. In the evolutionary tree the receptors in archegoniates (moss, spikemoss) keep aloof, attesting to the fact that three major types of receptors presumably emerged together with flowering plants but before their split into monocotyledonous and dicotyledonous plants [93].

**Fig. 6:**
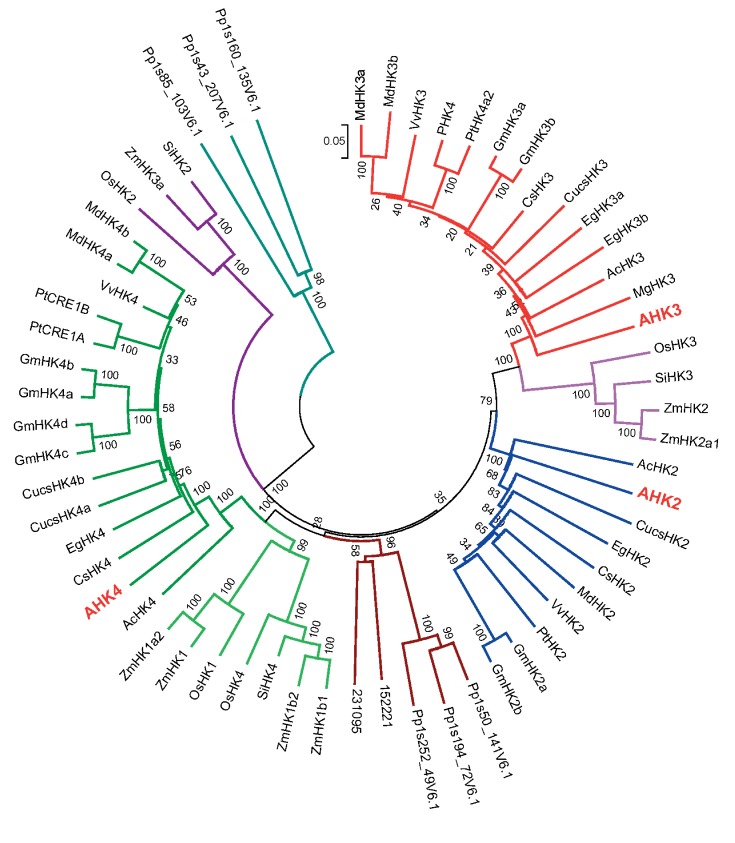
Phylogenetic analysis of cytokinin receptors. The sequence alignment was performed using the ClustalW program. The phylogenetic tree was built using the MEGA 5.05 software; the bootstrap analysis includes 1,000 replicates. Bootstrap supports for the individual branches are given as a percentage based on 1,000 bootstrap trials.

A broader phylogenetic analysis based on the sequenced genomes of 30 species of multicellular land plants provided further insight into the evolution of cytokinin receptors. Among the annotated genes 112 were revealed which encode proteins with a typical for cytokinin receptors domain organisation, including the CHASE domain, histidine kinase, and receiver domains ( *Fig. 6* ). The genes of these sensor histidine kinases are present in the genomes of all higher plants that have been sequenced. The number of sensor histidine kinases comprising the CHASE domain varies from one in potato *Solanum tuberosum * and the common monkey-flower *Mimulus guttatus * to eight in cultured soybean * Glycine max* . Several branches of closely related genes have been revealed in flowering plants via a phylogenetic analysis. Three branches corresponding to the Arabidopsis receptors АНК2, АНК3, and CRE1/AHK4 have turned out again to include the largest number of genes. A subdivision into groups of monocotyledonous and dicotyledonous orthologues has been observed in these branches. Moreover, certain small branches kept aloof; in particular, the group of monocotyledonous orthologues ZmHK3. In general, cytokinin receptors can be phylogenetically subdivided into three and four groups for dicotyledonous and monocotyledonous plants, respectively. The receptors of one plant species belonging to different groups are more similar to the group orthologues from other species than to each other within the same species. These receptor groups are non-identical in different plant species. As mentioned above, only one receptor belonging to the orthologues CRE1/AHK4 and AHK3, respectively, has been found in each of the dicotyledonous plants: potato and the common monkey-flower. If additional genes are identified in the genomes of these species, it might be that these species contain the other receptors, too. The StHK4 receptor from potato contains the phenylalanine residue instead of the conserved Tyr318. However, no direct evidence of a significant role played by this residue in receptor functioning has been offered [89]. It is of interest that tomato, a close relative of potato, carries the normal representatives of receptors belonging to all three major evolutionary branches. The CRE1/AHK4 orthologue is duplicated in the Leguminosae; four orthologues of CRE1/AHK4 (two in each duplication group) have been identified ( *Fig. 6* ). The two other branches contain two representatives of the soybean receptors, each. In lucerne *Medicago truncatula* the only orthologue of CRE1/AHK4 belongs to either one of the two duplication groups. The common bean *Phaseolus vulgaris* and *Lotus japonicus * have two representatives of the orthologue of CRE1/AHK4, each, but they do not have orthologues of AHK3 or AHK2, respectively. However, the highly conserved leucine in the PvHK4a from the common bean is substituted with tryptophan, which raises some doubt as to whether this protein can act as a cytokinin receptor. Few substitutions of the conserved amino acids have also been revealed in some other dicotyledonous species (sweet orange *Citrus sinensis* , cucumber *Cucumis sativus* , and cassave *Manihot esculenta* ). The common feature of all the dicotyledonous species (with the exception of the common monkey-flower) is the mandatory presence of orthologues of the CRE1/AHK4 receptor.

The monocotyledonous species rice and corn also has representatives of two evolutionary branches of receptors, the orthologues of AHK3 and AHK4. The AHK4 group can be divided into two subgroups, corresponding to ZmHK1a and ZmHK1b. In corn, two receptors belong to each of these groups/subgroups. However, the foxtail millet ( *Setaria italica* ) has no orthologues of CRE1/AHK4 in one of the subgroups (ZmHK1a), whereas sorghum ( *Sorghum bicolour* ) and *Brachypodium distachyon* have no orthologues of ZmHK3a ( *Fig. 6* ). Thus, all the known genomes of monocotyledonous plants encode at least one of the representatives of CRE1/AHK4 orthologues. It should be mentioned that the orthologues of CRE1/AHK4 are present in the sequenced genomes of almost all monocotyledonous plants in two versions. However, it is not improbable that this feature is typical only of the family Gramineae (for which the genomes have already been sequenced), whereas the other monocotyledonous families may contain a different number of CRE1/AHK4 isoforms. But in either case, orthologues of CRE1/AHK4 appear to be the most important cytokinin receptors in flowering plants at this point.

## CONCLUSIONS

Although the major plant hormones have been known since the mid-20th century, just in the past decades research in phytohormones has undergone a renaissance. This is due primarily to the elucidation of the molecular mechanism of their action on a cell; i.e., the discovery of the receptors and genes that encode them, and to the fact that it is now possible to clone and insert genes for cytokinin perception, biosynthesis and signal transduction, as well as to obtain targeted mutations [94–101]. The fundamentals for intracellular signalling of phytohormones are similar to those for the signalling of animal and human hormones. The role of receptors is to recognize the hormone; and some of the receptor properties are altered upon formation of the hormone-receptor complex, which results in signal transduction to the primary cellular target via the corresponding signal transduction system. Similarly to animals, the receptors in plant cells are mostly localized in two compartments: anchored on membranes or inside the nucleus (soluble receptors). The main cellular target for hormone signalling in plants and animals is a set of primary response genes, which is specific for each hormone. However, the molecular mechanisms of intracellular signal transduction have been found to be considerably different in plants and animals. Therefore the results of plant studies have significantly contributed to molecular hormonology as a field of science.

For cytokinin signalling, plants use a bacterial-type analogue of the two-component system of signal transduction, which presumably was borrowed from cyanobacteria [20, 24, 94, 102]. It is believed that the symbiosis of cyanobacteria and eukaryotic cells allowed plants to acquire chloroplasts and to use bacterial genes for this purposes [103, 104]. The landfall was a powerful stimulus for multicellular plants to form new hormonal regulation systems, including the cytokinin system. As animal cells contain no chloroplast-type organelles, animals lack the two-component signal transduction system, evidently because of the absence of symbiosis with the corresponding bacterial progenitors (cyanobacteria).

It is not by mere chance that the significant progress in revealing the molecular mechanisms of the action of phytohormones has been achieved in “post-genomic” 21st century. This was due to whole-plant genome sequencing, the Arabidopsis genome being the first study of the kind in 2000 [11]. As a result, Arabidopsis is as yet the only species whose cytokinin signal perception and transduction system has been thoroughly characterized. However, a host of questions remain unanswered even as regards this plant. In this respect, it is worth mentioning that studies devoted to the investigation of the cytokinin regulatory system are currently under way using various models and ultra-modern methods of molecular biology, hormonology, genetic engineering, bioinformatics, etc. The fact that we stand to soon witness new discoveries in this intriguing and promising field of natural sciences is beyond question. 

## Abbreviations

HK: histidine kinase
HP: phosphotransmitter
RR: response regulator
ER: endoplasmic reticulum
